# The dosimetric impact of stabilizing spinal implants in radiotherapy treatment planning with protons and photons: standard titanium alloy vs. radiolucent carbon‐fiber‐reinforced PEEK systems

**DOI:** 10.1002/acm2.12905

**Published:** 2020-05-31

**Authors:** Birgit S. Müller, Yu‐Mi Ryang, Markus Oechsner, Mathias Düsberg, Bernhard Meyer, Stephanie E. Combs, Jan J. Wilkens

**Affiliations:** ^1^ Department of Radiation Oncology Technical University of Munich, Klinikum rechts der Isar Munich Germany; ^2^ Department of Neurosurgery Technical University of Munich, Klinikum rechts der Isar Munich Germany; ^3^ Institute of Innovative Radiotherapy Helmholtz Zentrum München Neuherberg Germany

**Keywords:** carbon‐fibre‐reinforced PEEK, IMPT, spinal instrumentation, radiolucent spinal implants, radiation therapy, treatment planning

## Abstract

**Background:**

Throughout the last years, carbon‐fibre‐reinforced PEEK (CFP) pedicle screw systems were introduced to replace standard titanium alloy (Ti) implants for spinal instrumentation, promising improved radiotherapy (RT) treatment planning accuracy. We compared the dosimetric impact of both implants for intensity modulated proton (IMPT) and volumetric arc photon therapy (VMAT), with the focus on uncertainties in Hounsfield unit assignment of titanium alloy.

**Methods:**

Retrospective planning was performed on CT data of five patients with Ti and five with CFP implants. Carbon‐fibre‐reinforced PEEK systems comprised radiolucent pedicle screws with thin titanium‐coated regions and titanium tulips. For each patient, one IMPT and one VMAT plan were generated with a nominal relative stopping power (SP) (IMPT) and electron density (ρ) (VMAT) and recalculated onto the identical CT with increased and decreased SP or ρ by ±6% for the titanium components.

**Results:**

Recalculated VMAT dose distributions hardly deviated from the nominal plans for both screw types. IMPT plans resulted in more heterogeneous target coverage, measured by the standard deviation σ inside the target, which increased on average by 7.6 ± 2.3% (Ti) vs 3.4 ± 1.2% (CFP). Larger SPs lead to lower target minimum doses, lower SPs to higher dose maxima, with a more pronounced effect for Ti screws.

**Conclusions:**

While VMAT plans showed no relevant difference in dosimetric quality between both screw types, IMPT plans demonstrated the benefit of CFP screws through a smaller dosimetric impact of CT‐value uncertainties compared to Ti. Reducing metal components in implants will therefore improve dose calculation accuracy and lower the risk for tumor underdosage.

## 1 INTRODUCTION

Postoperative radiotherapy (RT) after spinal decompression and stabilization presents a common treatment combination in the surgical treatment of spinal tumors, which frequently faces challenges due to the presence of metallic implants. Based on computed tomography (CT) images, RT treatment planning, including both the contouring process of tumor and organs at risk (OAR) and the dose calculation, relies on accurate image information.^1^, ^2^ Dose calculation algorithms integrated in treatment planning systems (TPS) depend on correctly assigned Hounsfield units (HU), which are converted into relative stopping powers (SP) for proton RT and into relative electron densities (ρ) for photon RT.^3^, ^4^ Streak artifacts and the acquired CT values of the implant, which in case of highly absorbing materials tend to show saturation effects, contain uncertainties, that can lead to substantial erroneous calculated dose distributions.^1^ Moreover, developed for biological materials, clinical dose calculation algorithms are often insufficient to precisely model physical interactions associated with metallic implants.^5^, ^6^, ^7^

The relevance of these uncertainties depends on the treatment modality and the applied RT technique, such as type of particles, degree of intensity modulation, and beam geometry.^1^, ^5^ In general, (image) uncertainties are of greater relevance for proton compared to photon RT, where false CT numbers translate to errors of the estimated proton range^2^, ^8^ and can cause severe underdosage of the target volume.

Therefore, the application of protons in the presence of metallic hardware is controversially discussed. Dependent on the location, size of the implant, and institution, opinions range from “strict contraindication” to RT making use of clinical “workarounds” to improve treatment accuracy.^2^, ^5^, ^9^, ^10^, ^11^ The decision “pro” or “contra” proton treatment is challenging, considering on the one hand clinical studies which reported reduced tumor control rates for chordoma patients with titanium alloy implants compared to patients without surgical implants^9^, ^10^, ^11^ and on the other hand the chance for improved clinical outcome compared to photon RT. The advantageous OAR sparing and possibility to apply higher tumor doses^9^, ^10^, ^12^ indeed motivate to make use of the favorable particle properties.

For both proton and photon RT, the dosimetric impact of surgical implants as well as clinical measures to reduce associated treatment errors have been investigated broadly (e.g., Refs. [2, 5, 13]). The penetration of beams through implants should generally be avoided,^2^, ^4^, ^7^ which is — dependent on the tumor and implant location — not always possible. Additionally, the manual assignment of CT values for artifacts and implants is frequently suggested and part of clinical practice.^8^, ^9^, ^10^, ^11^, ^14^ While well‐investigated methods exist to determine calibration curves which relate HUs to SPs,^3^, ^4^, ^15^ the definition of metallic volumes is crucial and results in delineation and consequently dosimetric uncertainties.^1^, ^2^, ^5^, ^16^ Threshold‐based auto‐segmentation allows for delineation of CT regions within a specified range of HUs and promises hereby to reduce the subjectivity of the delineation process, but uncertainties in the CT scan due to the extension of the CT‐value range of the scanner^17^ and partial volume artifacts^18^ limit the accuracy of volume definition. Improvements of contouring and planning accuracy are expected by the application of MV‐CTs or dual energy CT for planning, as well as by advancing dose calculation and metal artifact reduction algorithms.^1^, ^6^, ^14^, ^19^, ^20^, ^21^, ^22^, ^23^, ^24^

Recent developments in neurosurgery, the introduction of carbon‐fiber‐reinforced Polyetheretherketone (CFR‐PEEK, or CFP for short) pedicle screw systems, may “solve” or at least improve the prescribed concerns in the future.^25^, ^26^, ^27^, ^28^ Dependent on the system and manufacturer, CFP systems feature a remarkably reduced amount of titanium or no metallic components, and thus decrease the impact of several correlated uncertainties. With its low atomic number, carbon‐based materials have favorable radiation properties compared to stainless steel and titanium alloy (Ti) implants and therefore have raised researcher's interest in the field of RT.^28^, ^29^, ^30^ Previously published measurements and simulations of proton and photon beam behavior through CFP compared to typical surgical metal implants underlined along with other investigations the promising properties of CFP implants.^28^, ^29^, ^30^ Although carbonic screws are also affected by HU uncertainties, as any component of the human body is, especially dense bones, associated uncertainties are considered to be remarkably smaller than those of high Z materials.

We expand the investigations on CFP implants by a retrospective planning study for intensity modulated proton (IMPT) and photon therapy (volumetric arc technique — VMAT), which examines the dosimetric impact of uncertainties in CT values of Ti in CFP pedicle screw systems, which comprise minor Ti components, compared to standard Ti systems. Uncertainties in HU assignment of Ti were simulated by varying SP and density, for IMPT and VMAT plans, respectively.

## 2 MATERIALS AND METHODS

### 2.A Patient information and contouring

The study is based on CT data of 10 patients, who were previously treated at our institute for spinal metastases with postoperative photon therapy after spinal decompression and stabilization surgery. All planning CTs, performed with a Siemens Somatom Emotion 16 Scanner (Siemens, Erlangen, Germany), at a tube voltage of 130 kV and variable mAs, had a resolution of 1 mm × 1 mm and a slice thickness of 3 mm. Five patients featured standard titanium alloy (Ti) implants and five carbon‐fibre‐reinforced PEEK (CFP) pedicle screw systems (Icotec, Altstätten, Switzerland).^26^ Carbon systems consisted of radiolucent non‐metallic CFP pedicle screws (diameter: 6.5 mm), CFP rods (diameter: 5.5 mm), a thin titanium coating in the pedicle area and titanium tulips. Ti screws measured a diameter of 6 or 7 mm and rods of 5.7 mm.

Planning target volumes (PTV) were located in the lumbar vertebral spine (9 patients) and the sacrum (1 patient). All patients received a monosegmental posterior instrumentation comprising of four pedicle screws (2 × 2) and 2 rods. Artifacts and metal components were generated systematically by applying threshold‐based auto‐segmentation, followed by manual adaption of the derived structures. Contouring was performed by the same staff member to minimize interpersonal variations. Since the number of slices which contained Ti components was comparably small compared to the whole PTV, a contour, referred to as “PTV local,” was defined as the PTV structure over the CT slices with the largest fraction of the screws. It consisted of 10 (2 × 5) PTV slices (Table 1) for all patients. PTV local reflects the dosimetric situation in proximity of the implants and allows for a better analysis of dosimetric changes inside the PTV for all patients. As critical organs varied between patients, a ring structure (“PTV ring”) of a 5 cm margin around the PTV extended by 5 mm was generated to evaluate dose to normal tissue (“PTV ring” = “PTV expanded by 5.5 cm” minus “PTV expanded by 0.5 cm”).

**Table 1:** Mean volumes and standard deviations of the delineated structures for titanium alloy (Ti) and carbon (CFP) systems.

Structure volume	Ti screws	CFP screws
	Mean ± SD (cm^3^)	Mean ± SD (cm^3^)
Metal	22.58 ± 5.36	9.02 ± 0.21
Artifacts	13.42 ± 6.36	3.06 ± 0.79
PTV	1151.4 ± 473.5	907.7 ± 228.3
PTV local	267.5 ± 117.9	276.4 ± 66.2

### 2.B Retrospective planning

Two initial plans were generated for each patient, one IMPT and one VMAT plan. IMPT plans were optimized and calculated in the research treatment planning system (TPS) matRad^31^ with a pencil beam dose calculation algorithm. VMAT plans were generated in Eclipse v13 (Varian Medical Systems Inc., Palo Alto, CA) with the AAA dose calculation algorithm.^32^

IMPT plans consisted of three beams with gantry angles of 150°, 180°, and 210°. The spot spacing within the PTV was 3 mm (water‐equivalent distance). The beam width of the generic proton machine was energy‐dependent, with a spot sigma of 5 mm at the nozzle exit and patient surface for lower energies (E < 70 MeV) down to 2.3 mm for the highest available energy (230 MeV). For the VMAT plans, we selected two full arcs, that is, from 181° to 179° and vice versa, with collimator angles of 10° and 350°. The beam energy was 6 MV and the leaf width 5 mm in the isocenter. No avoidance technique, such as avoidance structures or specific beam entrance limits, was utilized during the plan optimization.

The prescribed dose to the PTV was 30 Gy in 10 fractions for both RT techniques. Photon plans were normalized to the median in the PTV, proton plans resulted approximately normalized to the mean dose D_mean_ of the PTV after the optimization process. OARs were optimized dependent on the individual situation and tumor location. Doses are given in Gy, referring to RBE weighted dose for protons and to absolute absorbed dose for photons.

**Fig. 1:**
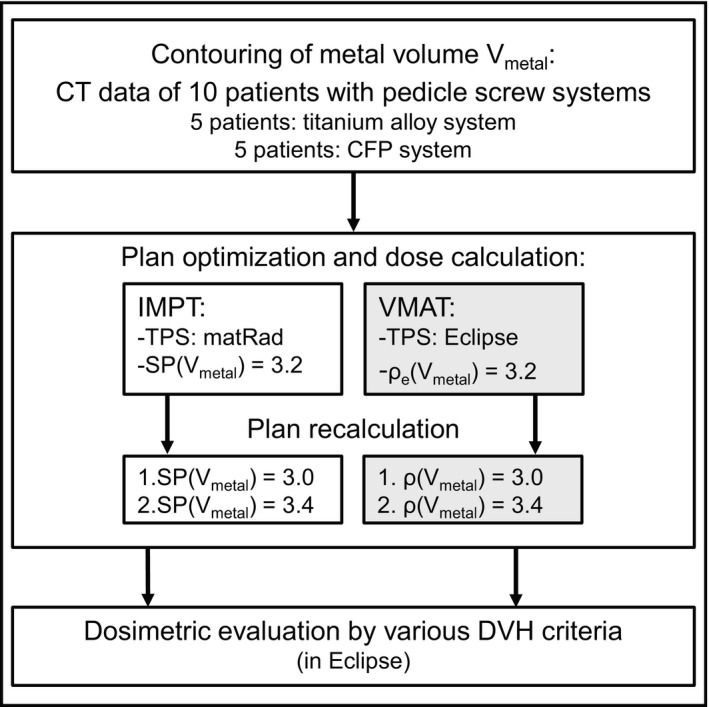
Planning study workflow.

To simulate uncertainties in the HU assignment of Ti components, varying relative stopping powers and electron densities were ascribed to the structure. Nominal plans were calculated with an SP and ρ of 3.2, for protons and photons, respectively. Both plans were recalculated onto the identical CT with increased and decreased SP or ρ by ±0.2 (~6%) to 3.0 and 3.4 (workflow, see Fig. 1).

### 2.C Plan evaluation

Plan quality was evaluated by target coverage, homogeneity, and by several dose volume histogram (DVH) criteria of the PTV, PTV local, and PTV ring. The maximum dose was analyzed by D2, the maximum dose received by 2% of the associated structure, and the minimum dose by D98, the minimum dose received by 98% of the structure. The standard deviation σ within the PTV served as a measure for target dose homogeneity.

## 3 RESULTS

### 3.A Volumes

**Fig. 2:**
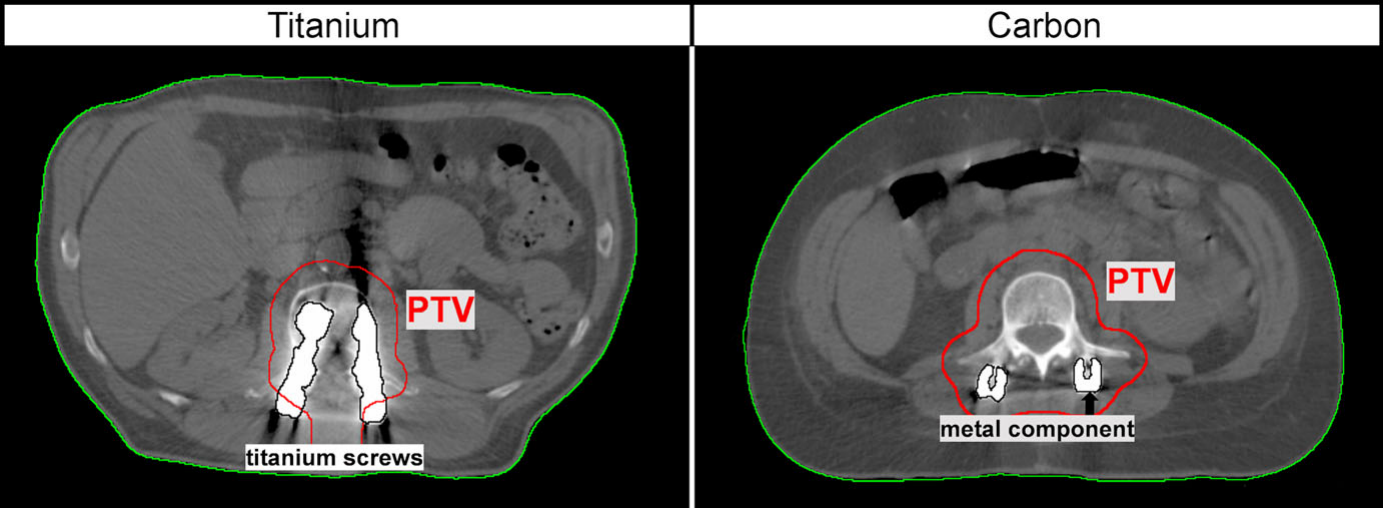
Computed tomography image and contours of the two implant types: titanium alloy (Ti, left) and carbon‐fibre‐reinforced PEEK (CFP, right). Carbon systems comprised non‐metallic carbon fibre reinforced PEEK screws and rods, thin titanium coatings in the pedicle area and titanium tulips. The derived contours indicate that perfect three‐dimensional rendering of the outline of the screw is hardly achievable and that corresponding structures comprise uncertainties.

The delineated structures of Ti components of both pedicle systems do not exactly reflect the real material outlines due to inaccurate representation in the CT scan; exemplary contours of one patient with CFP and one with titanium screws are presented in Fig. 2. Corresponding volumes of metallic components and image artifacts were smaller for CFP than for titanium alloy systems (Table 1).

### 3.B Nominal plans

**Fig. 3:**
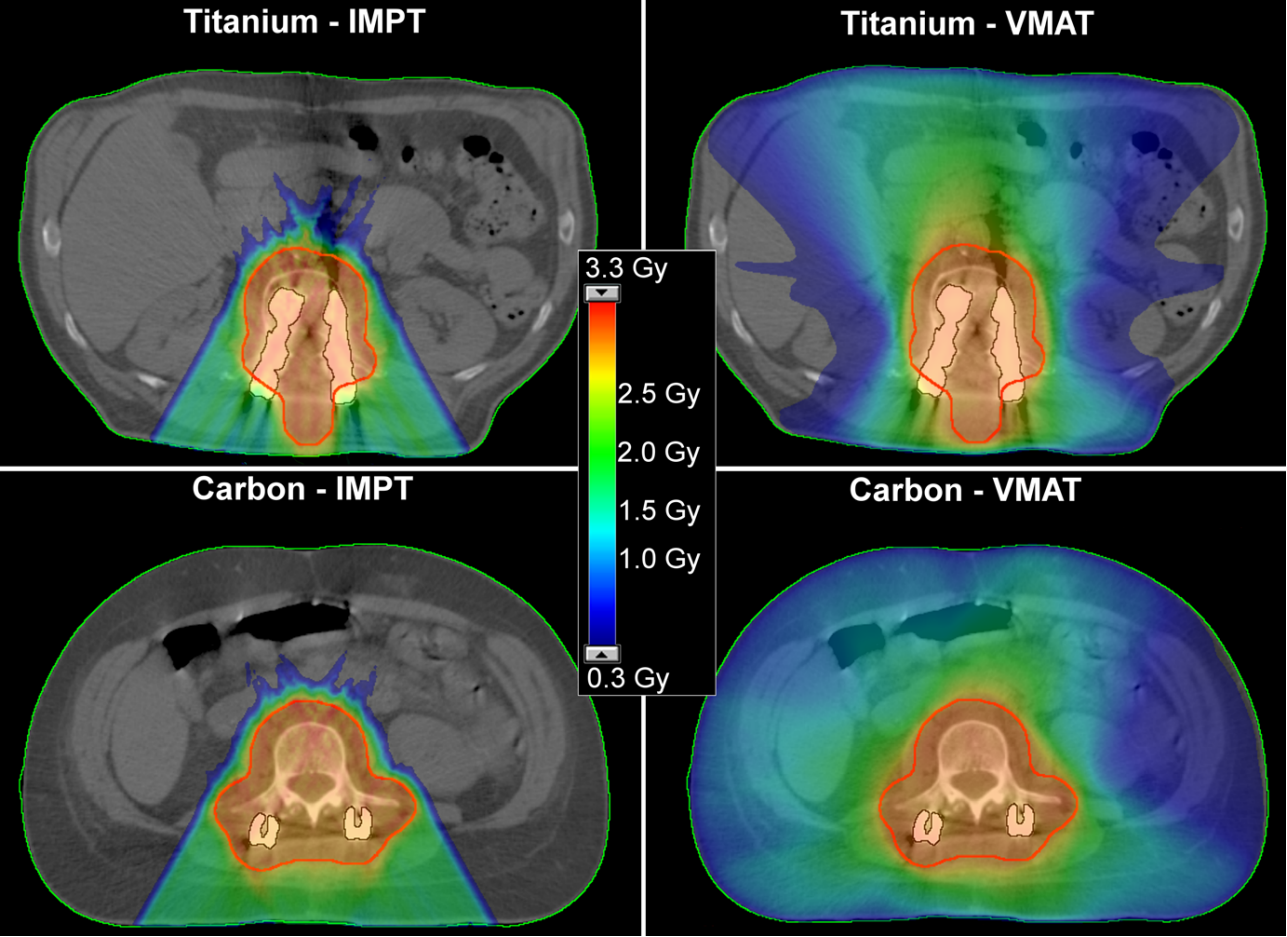
Exemplary initial plans, intensity modulated proton therapy (left) and volumetric arc photon therapy (right) of one patient with Ti (top) and one with carbon fibre reinforced PEEK pedicle screws (bottom). Optimization goals depended on the individual patient situation. The dose distribution of the presented Ti case was optimized with the focus on kidney sparing.

For both implants, dose to healthy tissue (Table 2) was smaller for IMPT compared to VMAT plans, with hardly any difference between the screw types (Fig. 3). Photon dose distributions presented a more homogeneous and slightly superior PTV coverage compared to protons for both implants.

**Table 2:** Dose to healthy tissue for both initial plan types and screw systems, quantified by the mean and D2 to the structure PTV ring (5 cm margin surrounding the PTV + 5 mm).

PTV ring	Ti screws		CFP screws	
	D2 ± SD (Gy)	D_mean_ ± SD (Gy)	D2 ± SD (Gy)	D_mean_ ± SD (Gy)
VMAT	2.48 ± 0.20	0.94 ± 0.15	2.44 ± 0.07	0.97 ± 0.06
IMPT	2.11 ± 0.11	0.37 ± 0.05	2.09 ± 0.05	0.38 ± 0.03

The comparison of CFP vs Ti for the initial VMAT plans by several dosimetric quality indicators (D2, D98, D95, σ of the PTV local) presented hardly any difference.

**Fig. 4:**
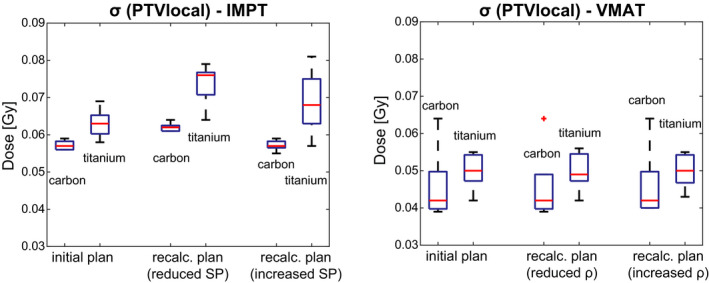
Intensity modulated proton therapy dose distributions of one exemplary Ti‐plan (top) and one exemplary carbon fibre reinforced PEEK‐plan (bottom); the initial plan, optimized with a relative SP for Ti of 3.2 (left), was recalculated onto the identical scan with a decreased SP (SP = 3.0) and an increased SP (SP = 3.4). Recalculations lead to larger overdosed and underdosed regions for Ti‐implants.

IMPT dose distributions showed reduced dose conformity and homogeneity in proximity to the Ti implants, caused by the large density gradient of penetrated materials (Fig. 3, left images of Fig. 4). Coverage and PTV dose homogeneity were superior for CFP than for Ti implants.

### 3.C Recalculated plans

**Fig. 5:**
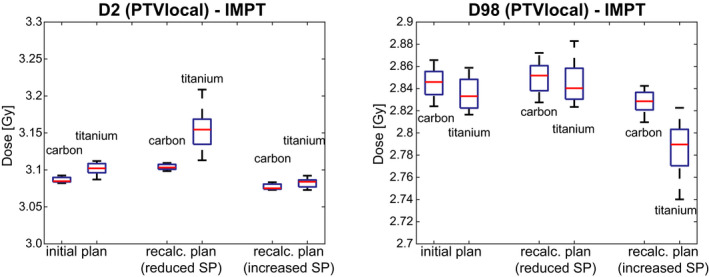
The standard deviation σ within the planning target volumes local as a measure for target dose homogeneity of the initial and both recalculated dose distributions for carbon fibre reinforced PEEK vs Ti implants. The red central line indicates the median of the evaluated criteria, the box represents the interquartile range (IR) and whiskers the most outer values within a range of 1.5 times the interquartile range. Outliers refer to a value larger than 1.5 × IR.

Recalculated VMAT dose distributions of both pedicle screw types were hardly influenced by the simulated HU deviations. Changes of DVH criteria were slightly larger for Ti, but all deviations were smaller than 1%, except for one case where the recalculated σ (PTV local) deviated by 2% from the nominal plan (Fig. 5).

The derived IMPT plans resulted in more heterogeneous PTV doses (Figs. 4 and 5), with a more pronounced effect for Ti screws; the standard deviation of the PTV local increased on average by Δσ(Ti)(PTV local) = 16.8% ± 4.7% and Δσ(CFP)(PTV local) = 8.4% ± 1.8% in plans with reduced SPs. For the recalculation with reduced SP, however, two exceptions were found for CFP and one for Ti, where the standard deviation decreased, that is, Δσ < 0 (Fig. 5).

**Fig. 6:**
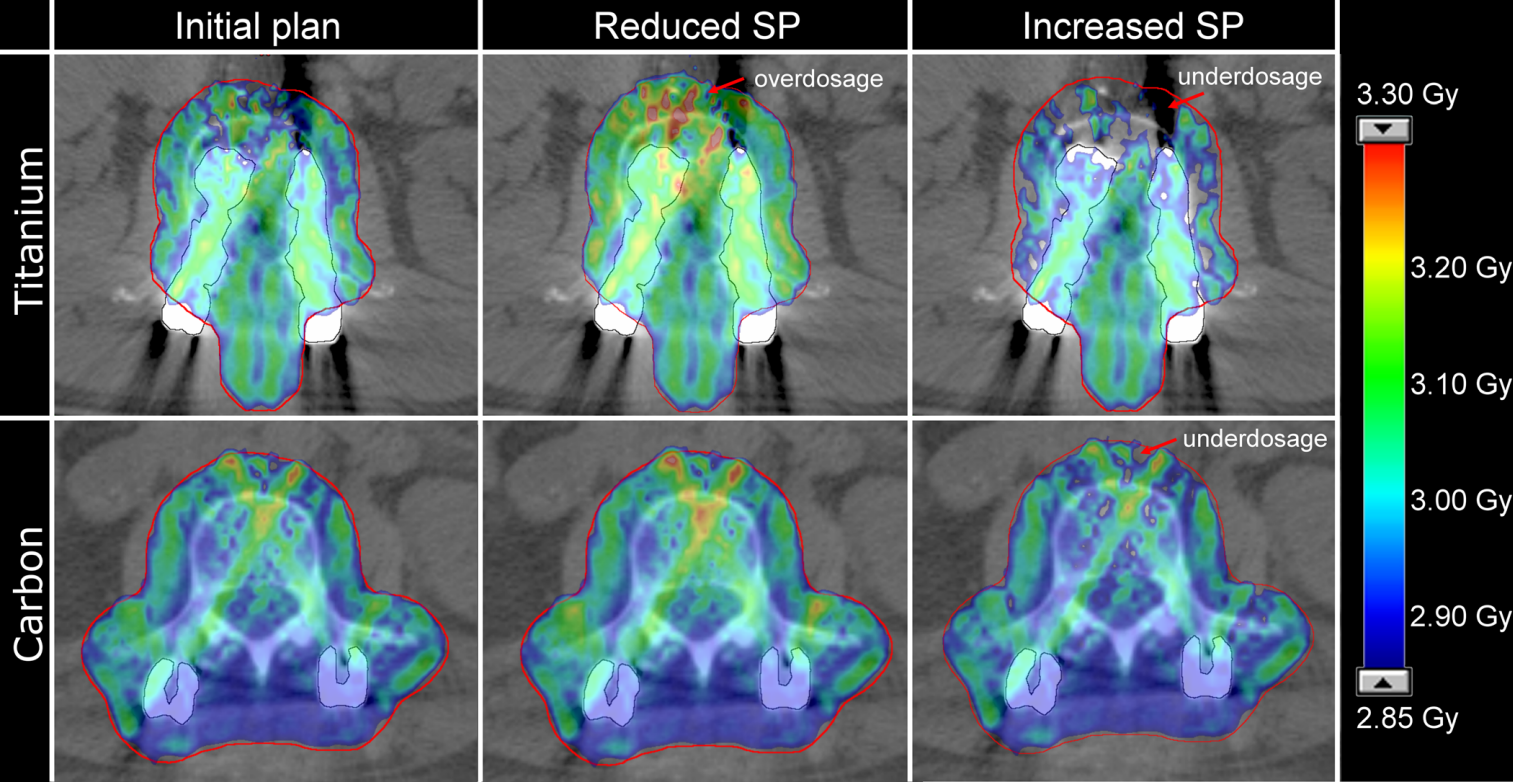
Maximum (D2, left) and minimum doses (D98, right) of the planning target volumes local of the initial and both recalculated Intensity modulated proton therapy plans for carbon fibre reinforced PEEK P vs titanium implants. The red line indicates the median of the evaluated criteria (details on boxplot presentation see Fig. 5).

**Fig. 7:**
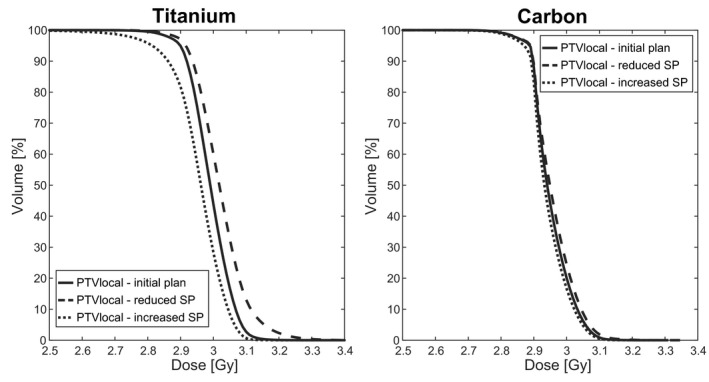
Dose volume histograms of the “planning traget volumes (PTV) local,” that is, the PTV region in proximity of the screw slices of one initial intensity modulated proton plan and two corresponding recalculated plans for Ti and CFP screws. Dose volume histogram correspond to the dose distributions of Fig. 4. The shape of the DVH curves of the target underlines the more stable coverage for C screws and indicates the regions of under‐ and overdosage by the reduced steepness for Ti.

Lower SPs, that is, the simulation of SP overestimation in the nominal plan, led to higher target maximum doses and increased SPs, that is, SP underestimation, to lower minimum doses (Figs. 6 and 7). The most extreme change of the minimum dose was measured by a decrease of ΔD98(Ti)(PTV local) = −4.2%. Regions of over and underdosage were caused by the metal components in both systems but were larger for Ti compared to CFP systems (Figs. 4 and 6).

## 4 DISCUSSION

Spinal stabilizing CFP pedicle screw systems promise to improve RT treatment planning accuracy compared to the prevalent titanium alloy pedicle screw systems, which among others cause dosimetric uncertainties through erroneous CT values that consequently translate into SP and ρ. With the elemental composition of CFP systems being similar to organic material and associated CT values not exceeding the commonly established scale of the CT scanner,^17^ corresponding potential HU uncertainties are remarkably smaller. For clinical proton RT, however, calibration curves of associated HUs and SPs have to be checked carefully.

The large number of patients who are affected by surgical implants and its potential for RT — especially for particle RT where dose distributions crucially depend on correct CT values — have made these neurosurgical developments to an active field of research.^26^, ^28^, ^29^, ^30^ Recent publications confirmed advantages of CFP over Ti implants for RT, among others through reduced dose perturbation effects.^28^, ^29^, ^30^

Our retrospective study complements previous research, by an RT treatment planning comparison of standard titanium alloy to CFP screw systems with titanium components for the commonly applied RT techniques, VMAT and IMPT. Potential dosimetric errors caused by CT value uncertainties of Ti in the planning CT were analyzed by simulating minor HU deviations.

Promising improvements by CFP compared to Ti screws were found for IMPT, whereas for VMAT the implants hardly affected the treatment plan quality. The difference in the dosimetric impact between VMAT and IMPT plans, and thus the greater relevance of the topic for protons than for conventional RT, is explained by the different physical particle properties and by the number of fields. VMAT dose distributions are blurred all over the body such that each beam angle has a minor impact on the whole dose distribution^1^ compared to the well‐defined beam angles in IMPT, which increase the influence of potential errors of the corresponding beam directions. Photon plans for different RT techniques, such as IMPT with well‐defined beam directions or avoided beam angle sectors in VMAT, may result in larger dosimetric consequences than observed here for VMAT plans with full arcs. Simulations performed outside the study showed that deviations in DVH criteria were, however, still remarkably smaller than for IMPT.

Not only were the dosimetric consequences of HU uncertainties in recalculated plans with Ti larger but also the nominal plan quality was worse than for CFP pedicle screws. Achieving the goal of a homogeneous dose coverage in CT slices with titanium was challenging. The great density gradient of the penetrated matter led to reduced target dose conformity and homogeneity. A large number of spots was placed along the traversed metallic parts of the implant due to its high density. To achieve dose coverage behind the implant (with respect to the proton beam direction), spots in that region required high spot weights, which simultaneously increased the dose in the entrance path before reaching the implant. This optimization conflict of finding a balance between over‐ and underdosage resulted in dose distributions that were rather a compromise than satisfying in these regions. Optimization difficulties were smaller for CFP screws, reflected in slightly superior initial plan qualities (Figs. 3 and 4). The selected beam set‐up comprised one field of 180° and two of slightly tilted angles, suboptimal traversing parts of the screw. To some degree, uncertainties and optimization difficulties are controllable via the beam angle selection,^1^, ^5^ but the planner is often limited in the choice by anatomy and geometry. Previous work also reported on degraded dose homogeneity associated with the presence of surgical implants.^8^, ^9^, ^12^ Rutz et al. further illustrated potential clinical consequences of hot spots, which required the reduction of the prescribed dose, and of cold spots as a potential risk for lower local control rate.^9^

Although limited by the number of patients, our results were consistent for the different screw types and RT techniques. Different TPSs and dose calculation algorithms served for the dose optimization and calculation of photons and protons. Since the intention of our study was not to compare between VMAT and IMPT but rather of the dosimetric impact for each individual technique, the different TPSs did not play a role for our conclusions.

One limitation, that has to be considered, is that our investigations present only one source of uncertainties in RT planning with titanium alloy implants, while the clinical situation reflects a combination of several potential errors. Each source of uncertainties should be investigated carefully and separately to estimate an overall risk of potential errors.

The observed dose changes caused by modified HU values were comparably small, and the reader might question the clinical relevance of the measured differences. Indeed, for spinal metastases and the prescribed dose of 30 Gy, the observed dosimetric influence may not have any clinical impact.

For chordoma and chondrosarcoma patients, however, proton therapy is a common treatment choice to achieve high tumor doses, as required for their treatment due to the low radiosensitivity while adhering to tolerance doses of neurological structures.^9^, ^10^, ^11^, ^33^, ^34^ By extrapolating our results to these higher doses resulting differences of one or two Gray over all fractions may decide over tumor control. Considering that the examined HU variations of titanium alloy only present one aspect of several sources of errors, these dosimetric uncertainties should not be neglected.

The definition of the implant contours, that is, what should be defined as Ti (very high ρ/SP) and what is organic tissue (low ρ/SP), is critical. Although utilizing supportive automatic contouring tools, uncertainties remain. Partial volume artifacts,^18^ particularly on the edge between tissue and implant make an exact delineation impossible (see Fig. 2). Larger delineation uncertainties were observed for Ti compared to CFP screw systems (see Table 1), which translate to larger uncertainties in the recalculated dose distributions. With respect to our study, this aspect presents a potential weakness, as dosimetric consequences of potential erroneous outlines of the Ti component were not investigated.

At the same time, these concerns indicate that the contouring process of implant material may be a source of uncertainties in the broadly applied clinical workaround of manual ρ/SP assignment. While uncertainties in ρ/SP are comparably well controllable, that is, ρ/SP are well determined,^4^ CT resolution is limited and delineation is not perfect, which makes the assignment to the correct volume a difficult task. Therefore, the delineation process of metal implants, the basis of this manual assignment, should be considered as a critical source of errors and could be subject of future investigation.

Different screw systems are available, ranging from pure metallic compositions to complete metal‐free and combined material systems.^26^, ^27^ The choice of the implant is typically driven by the surgeon and disposability of a clinic; advantages and disadvantages from the surgical perspective exceed the scope of this work. For the here investigated CFP systems with remaining metallic components, the advantage of CFP is limited, as perturbations of the dose distributions remain. Whether the potential errors and the risk for under‐ or overdosage are small enough and advantages in clinical outcome considered large enough to justify the application of proton therapy is for sure situation‐dependent and has to be weighed individually. There are certainly scenarios where these CFP screw systems are no obstacle for proton therapy at all. If the target is located adjacent to the CFP screws and titanium components lie outside the treatment area, beam angles can be selected without penetrating the titanium fraction.

Generally, CFP pedicle screw systems present a reduction of metal compared to their metallic analogues such that obviously associated uncertainties will be reduced. Less artifacts and dose calculation errors, due to inaccurate simulated particle transport in metal, will improve dose calculations. Although not investigated here, the contouring process of targets and critical structures is expected to gain in precision from better visualization, with a particular benefit for more complex cases. Mastella et al. demonstrated the higher image quality by measuring remarkably smaller variations of CT values of the same region in proximity of carbon screws.^30^ Moreover, they noted improved planning accuracy for proton therapy with CFP implants, concluding that with carbon implants patients have greater flexibility for future RT treatments — a consideration with which we completely agree with. Weighing aspects of the benefit of particle therapy due to higher applicable doses against uncertainties caused by metallic implants and the correlated risk for local failure or OAR complications, as well as the search for methods to reduce uncertainties and their clinical risks, could be of the past if metallic implants were fully exchanged by carbon‐based materials, provided that the clinical outcome from the neurosurgical perspective is maintained.

## 5 CONCLUSION

VMAT plan quality was hardly affected by the surgical implants and by the simulated HU uncertainties. While the clinical relevance of the investigated uncertainties is considered to be negligible for this technique, IMPT plans clearly demonstrated advantages of CFP surgical instrumentation. More heterogeneous target coverage in proximity of metallic components of all IMPT plans and greater dosimetric consequences of HU uncertainties of titanium alloy proved the superiority of RT planning with CFP screw systems. CFP instruments arise hope to enable treatments with particles of cases which were so far excluded from particle RT due to their metallic hardware and correlated uncertainties. Due to the remaining titanium fraction, which still leads to degradation of the dose distribution, the benefit of CFP screws is not fully exploited in the examined screw systems. Generally, further reduction of metal components in pedicle systems will further improve dose calculation accuracy and the contouring process through a higher image quality. This gain in dosimetric quality will consequently reduce the risk of tumor underdosage and overdosage of critical OARs.

## CONFLICT OF INTEREST

BSM, MO, MD, SEC, JJW: no conflicts of interest. YMR: speaker/teacher and consultant for Icotec. BM: consultant for Icotec. The Department of Neurosurgery received research funds from Icotec.
